# Bouncing of an ellipsoidal drop on a superhydrophobic surface

**DOI:** 10.1038/s41598-017-18017-2

**Published:** 2017-12-18

**Authors:** Sungchan Yun

**Affiliations:** Department of Mechanical Engineering, Korea National University of Transportation, Chungju, 27469 Republic of Korea

## Abstract

Drop impact on superhydrophobic surfaces has received significant attention because of the advantages of self-cleaning and anti-icing attained by minimum contact time with the surface. Drop hydrodynamics is generally assumed to be axisymmetric, and the contact time is still bounded below by a theoretical Rayleigh limit. In this study, we report an ellipsoidal drop impact on a superhydrophobic surface to demonstrate an efficient way to reduce the contact time and suppress the bounce magnitude by breaking the symmetry. The outcome of the bounce is characterized in terms of a geometric aspect ratio (AR) and Weber number of the drop by comparing the dynamics with a spherical drop. The experimental result shows that the bouncing of the ellipsoidal drop can reduce the contact time and maximum bounce height below the spherical one by at least 30% and 60%, respectively. The exceptional rim dynamics at high AR produces a liquid alignment along the principal direction, leading to the symmetry breaking in the mass and momentum distribution and the subsequent fast drop detachment, which is quantitatively rationalized by the numerical study. The distinct features of the ellipsoidal drop impact will provide an insight into shape-dependent dynamics and open up new opportunities for self-cleaning and anti-icing strategies.

## Introduction

Controlling the outcomes of drop impact is important for a wide variety of scientific and industrial fields, including inkjet printing, pesticide deposition, and chip cooling^1–3^. On superhydrophobic surfaces, the drop impact has received significant attention because of the benefits of staying dry, self-cleaning, and anti-icing^4–11^. In general, drops hitting the surfaces can bounce off rapidly due to the low wetting hysteresis at the liquid–solid interface^12^, and the contact time at the interface depends on various factors, such as inertia–capillarity, internal dissipation, and liquid-solid interactions^3,4,13–15^. The hydrodynamics of bouncing drops is governed by complicated mass, momentum and energy transfer between liquid and solid, providing a convincing relationship where the contact time is proportional to the inertia–capillary time scale, *τ* ~ (*ρD* ^3^/*σ*)^1/2^, where *ρ*, *D*, and *σ* are the density, equilibrium diameter, and interfacial tension, respectively^4,16,17^. Thus, the emphases of current trends mainly focus on breaking through in a reduction in the contact time below the theoretical limit.

Conventional drop impact dynamics is assumed to be axisymmetric during bouncing. Thus, many attempts have been made to explore strategies to improve the classical contact time bounded below by the limit. Bird *et al*. showed that breaking the symmetry resulted in a reduced contact time below the theoretical limit by introducing a macro–ridge structure with a submillimeter size on the surface^18^. The fast retraction along the ridge induced the lifting off of drop center with fragmentation, and the reduction in contact time depended on the impact velocity in a step-like behavior^19^. Liu *et al*. reported that a superhydrophobic surface decorated with micro/nano structures allowed a counter-intuitive bounce of the drop leaving the surface with shortened resident time^20,21^. The drop reached the maximum spreading diameter and then instantly bounced off the surface in a pancake shape without retraction. Subsequently, Liu *et al*. demonstrated that drops impacting on cylinders, of which curvature is comparable to the drop size, can accelerate the detachment speed by reducing its mass with symmetry-breaking flows^22^. The study emphasized that the rapid drop detachment demands a large driving force in central film and a small inertia of the rim at the periphery of the film^22,23^. Recently, Shen *et al*. found that drops impacting on a convex surface with a dome shape rapidly detach from the surface by evolving into an annulus shape where the inner and external rims have a high retracting velocity^24^. The idea for all these studies is to utilize surface morphologies that could create fresh regimes to further reduce the contact time^25,26^.

Based on the shape-dependent drop impact reported by our group, the bounce magnitude can be efficiently handled when the drop that is deformed into ellipsoidal shapes hits a hydrophobic surface and a heated surface that can generate Leidenfrost drops^27–29^. The ability arises from the break-up of the axisymmetry, allowing a portion of the axial kinetic energy (KE) to be converted into non-axial KE due to an induced transverse shape oscillation. Distinctively, the method can alter the impact behavior substantially without fragmenting the drop or modifying either the chemical composition of the liquid or the solid surface.

The previous study reporting the anti-rebound of the ellipsoidal drop on the hydrophobic surface presented a dominant role of the geometric aspect ratio (AR) in the rebound/deposition outcomes, although the study did not investigate the characteristics of the bouncing dynamics, such as the contact time and bounce height^28^. Other study introducing the bounce control of the Leidenfrost drop on the heated surface with ellipsoidal shaping confirmed the feasibility of a reduction in the contact time and bounce magnitude, which is considered a drop impact on a perfect non-wetting surface^29^. The numerical model demonstrated the reduction, but slight discrepancies between the model and experiment still existed because the model did not consider thermodynamic effects related to the formation of a vapor layer under the liquid and a hole due to the rupture of the lamella^29^. The model is more accurate and suitable in the current work studying the bouncing dynamics on the superhydrophobic surface at room temperature. In addition, the previous study of the Leidenfrost drop did not have a clear explanation for the origin of the reduction in the contact time. In the current work, we first report the bouncing of the ellipsoidal drop on the superhydrophobic surface and interpret the underlying principle of how the shape-dependent impact affects the reduction in the contact time.

The drop impact is represented by several physical quantities including drop size and impact velocity (*U*), which can be organized into some dimensionless numbers: the Reynolds number (Re = *ρDU*/*μ*) indicating a relative ratio of the inertial force and the viscous force, the Weber number (We = *ρDU* ^2^/*σ*) representing a relative ratio of the inertial force to the surface tension, and the Ohnesorge number (Oh = *μ*/(*ρσD*)^1/2^) denoting a relative ratio of the viscous force to the inertial and capillary forces. The parameters used in this study (*D* = 1.97 mm; *U* = 0.6–1.1 m s^–1^) yielded the following results: Re = 1200–2200, We = 9–33, and Oh = 0.0026 for the water drop at room temperature. This regime suggests that the viscous force is negligible in drop impact, whereas the inertial and capillary forces are highly important in drop impact.

We define the geometric aspect ratio of the ellipsoidal drop (AR = 1.0–1.8) as a ratio of the major axis to the minor axis in the horizontal plane obtained just before impact. In addition, the normalized contact width, height, and maximum bounce height by *D* are denoted as *w*, *h*, and *h*_*m*_, respectively. The normalized mass per unit length and the momentum by the initial value of the spherical case are represented as *M* and *p*, respectively. The minor and major axes of the ellipsoids are represented as the principal directions of *x* and *y* in drop impact; the *x*-direction is denoted as the axis with which the liquid is aligned during retraction.

In this study, we present an ellipsoidal drop impact on a (macroscopically) flat superhydrophobic surface showing a reduction in the contact time and suppression of the bounce magnitude and focus on the effect of the AR on the bouncing dynamics. The spherical and ellipsoidal drop dynamics were captured using high-speed cameras and analyzed with an image processing, which is characterized in terms of the contact width and the height as a function of AR and We. The numerical simulation employing a volume-of-fluid (VOF) method suggests that extraordinary rim dynamics result in the symmetry breakage of the mass and momentum distribution, thereby yielding a striking contrast between the spherical and ellipsoidal drops. We discuss the drop detachment speed and the maximum bounce height based on the momentum transfer between the principal (horizontal) and vertical directions.

## Results and Discussion

**Figure 1:**
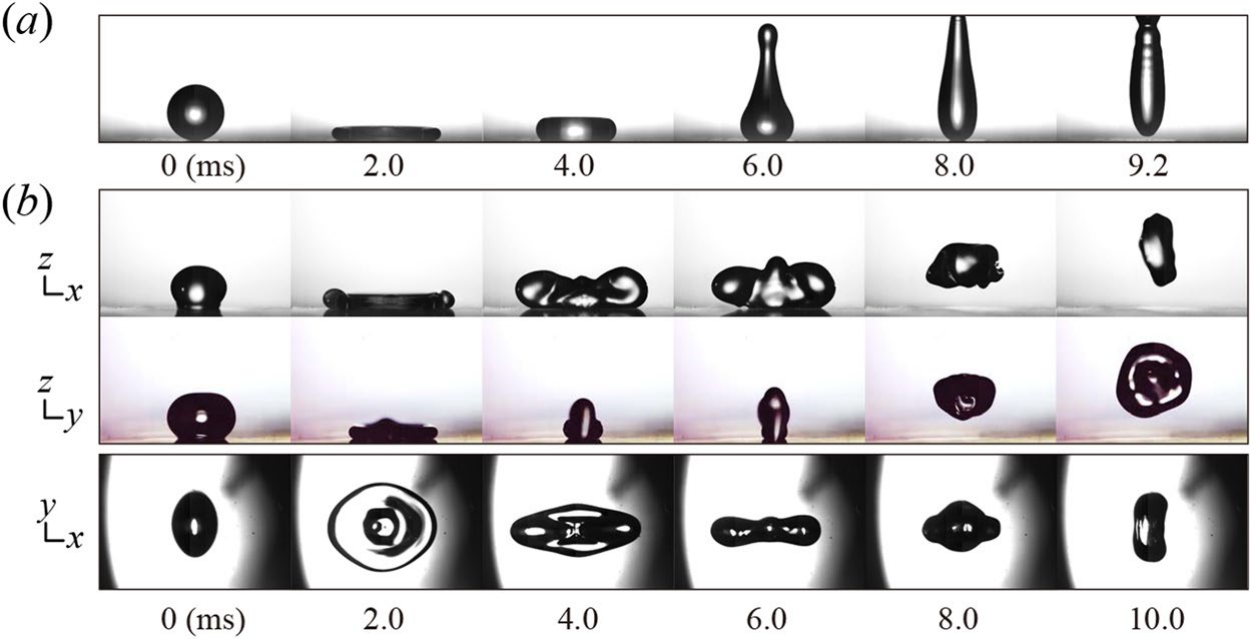
Impacting and bouncing behaviors of a water drop (diameter *D* = 1.97 mm; impact velocity *U* = 1.0 m s^−1^; We = 27). (**a**) High-speed images of a spherical drop show that the drop bounces off the surface at 9.2 ms. (**b**) Side-view images synchronized with each other and plane-view images of ellipsoidal drops (AR = 1.45) demonstrate that the bouncing behavior is dramatically altered, leading to a reduction in the contact time and bounce height.

Figure 1a and b show that the impacting and bouncing behaviors of a typical spherical drop and an ellipsoidal drop, respectively. The spherical drop continued to spread in a nearly axisymmetrical manner, and the retracting rim thickened, moving rapidly toward the center of the liquid that remained stationary on the surface. The drop finally lifted off the surface after 9.2 ms (=0.90(*ρD* ^3^/*σ*)^1/2^) and soared into the air; the experiments of Richard *et al*. reveal the prefactor close to 0.91^4^. The symmetric retraction and large inertia of the rim would limit the fast drop detachment^22^. Distinct from the typical case, the ellipsoidal drop showed a non-axisymmetric morphology of shape throughout the impact. We obtained the synchronized side-view and plane-view images to scrutinize the bouncing dynamics. The elliptical shape at the moment of impact allowed a difference in maximal spreading widths between the principal directions of *x* and *y*, thereby leading to the formation of the liquid alignment along the *x*-direction, as indicated by 4.0 ms in Fig. 1b. The liquid started to be released and retracted along the direction by a capillary force at 6.0 ms and bounced off the surface at 7.0 ms (=0.68(*ρD* ^3^/*σ*)^1/2^). The liquid alignment formed at the retracting state served as an initial shape to trigger the drop oscillation alternating between oblate (shown at 10 ms) and prolate ellipsoids that were nearly axisymmetric with respect to the *x*-direction after bouncing. The exceptional process in the ellipsoidal drop impact from the alignment to the oscillation yields the non-axial components of KE (principal directions) to be exchanged between the principal directions, thereby reducing the axial (vertical) KE.

**Figure 2:**
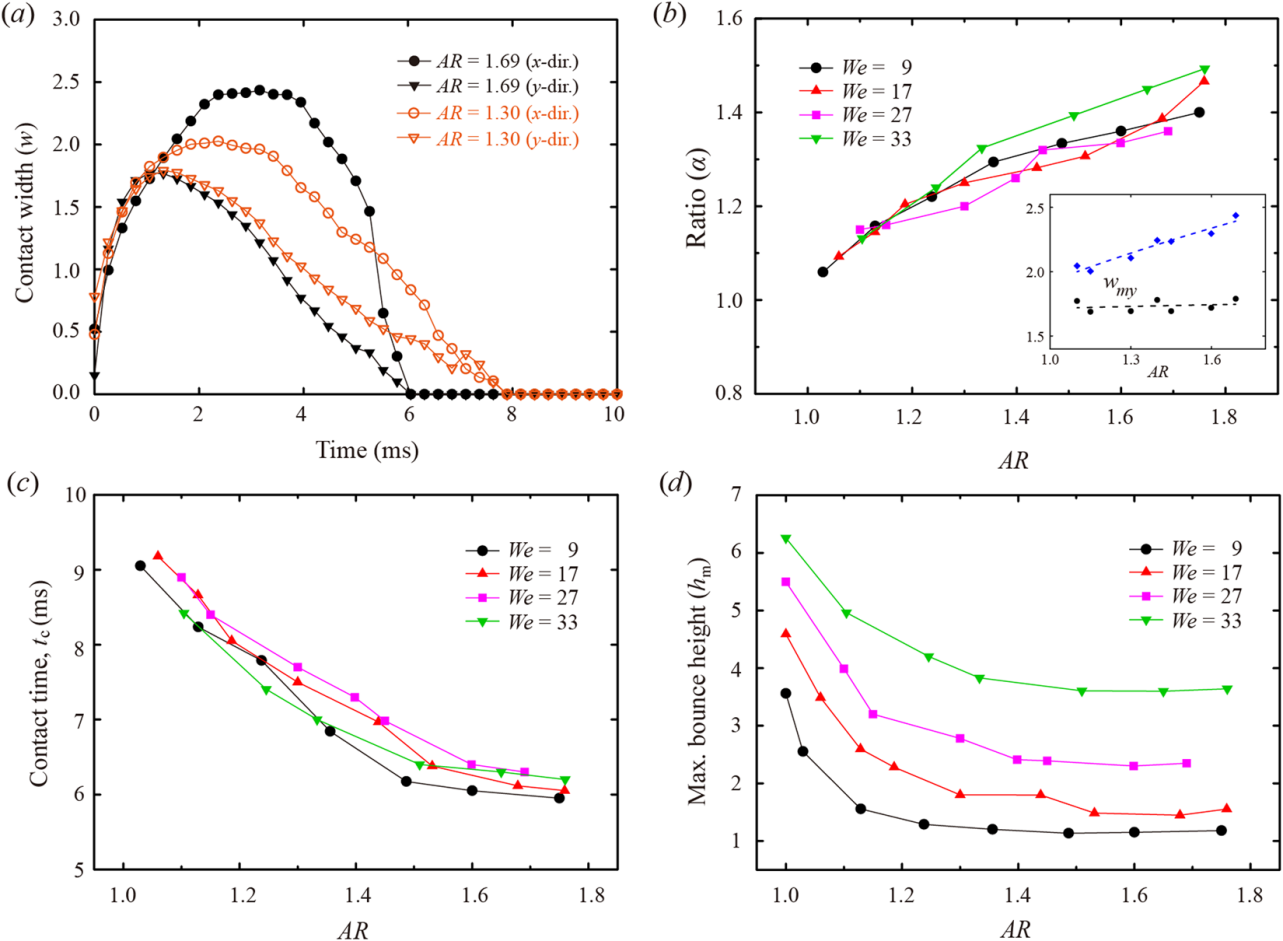
Bouncing of the ellipsoidal drop. (**a**) Time evolution of the normalized contact widths of drops in the principal directions at the same impact velocity (We = 27). (**b**) The ratio of the maximal spreading widths between the principal directions (*α* ≡ *w*_*mx*_/*w*_*my*_) as a function of AR under different We. The inset represents the variations of the maximal spreading widths observed at We = 27, which shows that the maximal extensions of the *y*-direction remain nearly constant, compared with those of the *x*-direction. (**c**,**d**) The normalized contact time (*t*_*c*_) and the maximal bounce height (*h*_*m*_) as a function of AR under different We. *t*_*c*_ and *h*_*m*_ decay with increasing AR and become almost saturated once AR is almost greater than 1.5. The normalized contact width and maximum bounce height by *D* are denoted as *w* and *h*_*m*_, respectively.

The bouncing features of the ellipsoidal drop were characterized in terms of the contact width in the principal directions, maximal bounce height, and contact time for varying ARs and impact velocities. Figure 2a shows the temporal evolution of the normalized contact widths in the principal directions for different ARs at the same impact velocity. As the AR increased, the difference in the maximal spreading widths between the principal directions increased, which implies that the liquid alignment is well developed for high AR. The contact time is significantly affected by the AR, and the fast detachment of the bouncing drop can be achieved for the high AR. The reduced contact time by roughly 2.0 ms between two ARs is shown in Fig. 2a. Figure 2b denotes the ratio of the maximal spreading width between the principal directions (*α ≡ w*_*mx*_/*w*_*my*_) as a function of AR under different We. The plot shows that an increase in AR gives rise to a larger α because the maximal spreading width of the *x*-direction indicates a strong increase with the AR, whereas that of the *y*-direction remains nearly constant with increasing AR, as described in the inset of Fig. 2b.

The contact time (*t*_*c*_) and maximal bounce height (*h*_*m*_) are plotted in Fig. 2(c,d) as a function of AR and We. The plots indicate that the *t*_*c*_ and *h*_*m*_ decay with increasing AR and become saturated once AR is almost greater than 1.5 at the same We. For AR = 1.60 and We = 27, *t*_*c*_ and *h*_*m*_ were reduced below those of the spherical case by nearly 30% and 58%, respectively. We also found the only slight dependence of the *t*_*c*_ on We as shown in Fig. 2c, which agrees with the result that the contact time is independent of the impact velocity in the inertia–capillary regime, proportional to (*ρD* ^3^/*σ*)^1/2^ ^4^.

The liquid alignment observed for high AR produces the non-axial KE due to the shape oscillation, which then mitigates the bounce magnitude. We suppose that the dependence of the *h*_*m*_ on AR is closely related to the ratio of the maximum non-axial KE to the initial KE in the retraction process, based on the previous numerical model of the Leidenfrost drop^29^. The ratio means a maximum asymmetry in the KE that the drop can hold, showing that the value is proportional to the AR and increased up to approximately 0.4 at AR ~1.5, but it has no significant variation beyond the AR. This finding is consistent with the experimental observations of *t*_*c*_ and *h*_*m*_. In addition, the model reveals that the ratio is only slightly affected by a variation in We. As the absolute axial KE driving the drop bouncing increases with the We, the maximal bounce height is basically proportional to We at the same AR, as shown in Fig. 2d.

One might suggest that the ellipsoidal shape oscillation before impact can affect the bouncing dynamics on the superhydrophobic surface. However, the effect is almost negligible in the regime of this study. In other words, when the drop collides with the surface, the shape is assumed to not change with a fixed AR because the time for the drop to touch the surface, namely, spreading time (*τ*_*s*_ ~ *D/U*), is much shorter than a period of the oscillation (*τ*_*o*_ ~ (*ρD* ^3^/*σ*)^1/2^). The ratio of the time scales can be given as *τ*_*s*_ */τ*_*o*_ ~ We^–1/2^, which means that the shape oscillation weakly affects the bouncing dynamics for high We. This finding is confirmed by a demonstration that the impact behavior of the slightly deformed ellipsoidal drop with the shape oscillation (AR = 1.01) is fairly similar to that of the spherical drop in the experiment.

**Figure 3:**
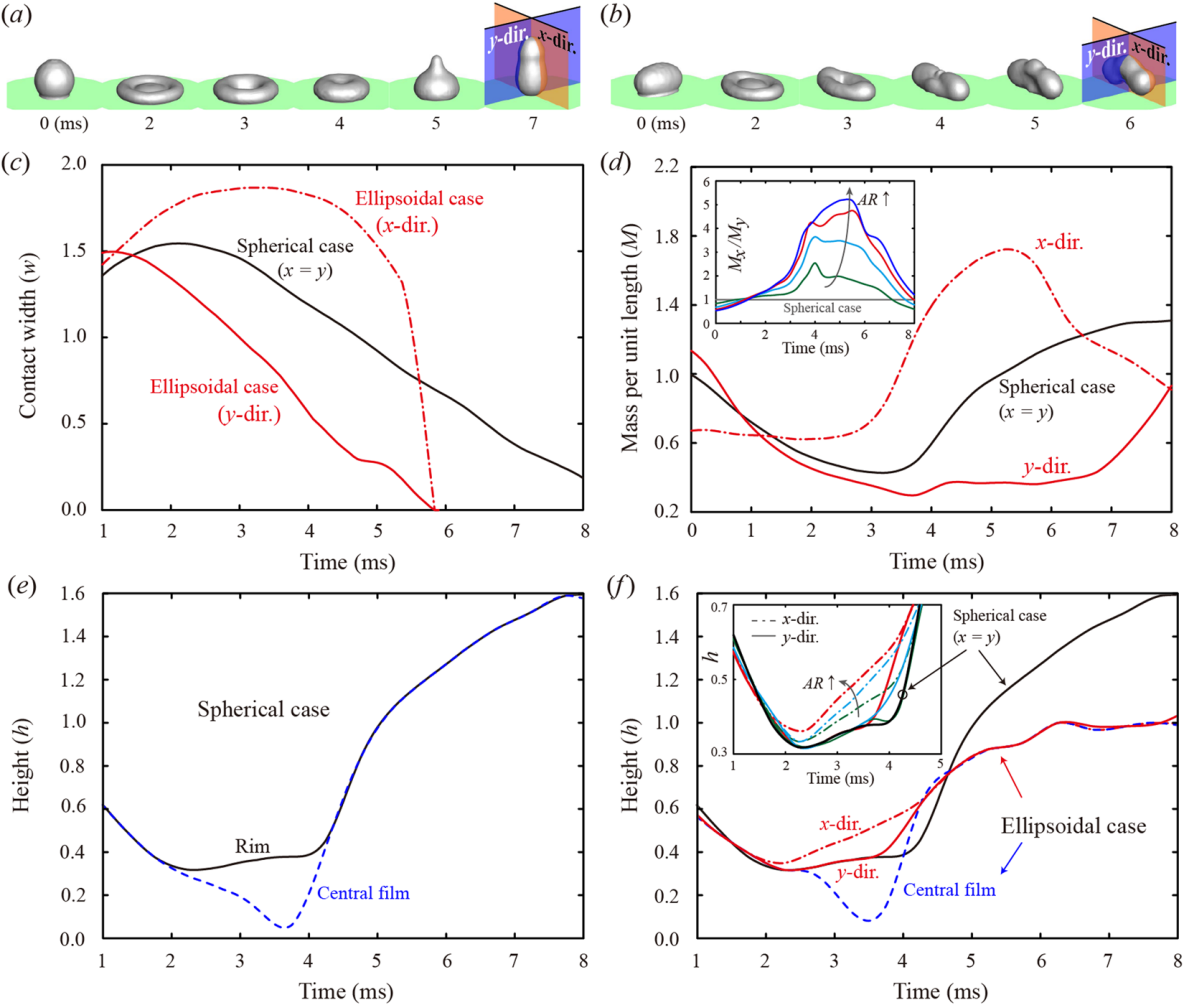
Effects of AR on the bouncing dynamics of the drop in the numerical simulation. (**a**,**b**) Time evolution images of the spherical drop and ellipsoidal drop for AR = 1.62 at We = 17, obtained by the numerical simulation. The cross-sectional planes on the *x*- and *y*-directions including the center of the drop are indicated in the last images. (**c**,**d**) Temporal evolution of the normalized contact widths and the normalized mass per unit length (*M*) along the cross-sectional planes described in (**a**,**b**) for AR = 1.62. The mass per unit length is scaled by that of the spherical case at the initial state (*t* = 0). The dash-dot line indicates the *x*-direction, and the solid line indicates the *y*-direction. The inset of (**d**) represents the temporal variation of the ratio of *M* between the *x*- and *y*-directions for AR = 1.17 (green), 1.46 (cyan), 1.62 (red), and 1.76 (blue). (**e**,**f**) Time variation of the normalized heights of the rim and the central film for the spherical drop and the ellipsoidal drop of AR = 1.62 in the principal directions based on the simulation. The normalized height by *D* is denoted as *h*. The inset of (**f**) represents the time variation of the rim for several ARs. For comparison, the spherical case of the rim (black line) is included, which shows that the rim height of the *x*-direction of the ellipsoidal drop increases with AR, whereas that of the *y*-direction shows no significant variation compared with the spherical case.

To interpret the fast drop detachment of the ellipsoidal drop, we scrutinized the effect of AR on the bouncing dynamics using the numerical simulation. Figure 3a and b show snapshots of the time evolution of the spherical drop and ellipsoidal drop for AR = 1.62, respectively. The variations of the contact widths of the drops are plotted in Fig. 3c. For the ellipsoidal case, the dash-dot line indicates the *x*-direction, and the solid line indicates the *y*-direction. The ellipsoidal drop of the *y*-direction and the spherical one reach the maximum spreading widths (*w*_*m*_ ~ 1.5) and start to retract at nearly 1 and 2 ms, respectively. The ellipsoidal drop of the *x*-direction, however, continues to spread and reaches the maximal extension that remains almost constant for a few milliseconds. The liquid rims of the spherical drop move inward and gather at the center to elongate the drop vertically, whereas those of the ellipsoidal drop show the apparent break-up of the symmetry and the consequent formation of the larger rims along the *x*-direction than along the *y*-direction, as shown in Fig. 3(a,b). The remarkable rim dynamics of the ellipsoidal case drives the discrepancy in mass distribution between the principal directions, which is closely related to the prompt drop detachment.

The mass distribution along the directions is plotted in Fig. 3d, where *M* represents the normalized mass per unit length along the cross-sectional planes by that of the spherical case at *t* = 0 (i.e., *M* = 1.0 for the spherical case); the planes include the drop center, as indicated in Fig. 3(a,b). The *M* of the *x*-direction (*M*_*x*_) is almost unchanged until nearly 3.0 ms but abruptly rises thereafter because the merging of the rims starts from both ends along the *x*-direction, as shown at 3.0 ms in Fig. 3b. Similar to the “zip-up” process, the merging continues to the drop center along the *x*-direction as shown at 4.0 ms, whereas *w*_*x*_ remains almost constant. By contrast, the *M* of the *y*-direction (*M*_*y*_) continually decays until nearly 3 ms and stays below 0.4 until the drop bounces off, which induces a small inertia of rims to be merged. The mass distribution in the principal directions means that the extensive mass transfer from the *y*-direction to the *x*-direction occurs until nearly 3 ms through a main flow to the *x*-direction, which causes the maximum ratio (*M*_*x*_ */M*_*y*_) to be approximately 5.0 at the highest AR, as shown in the inset of Fig. 3d. The maximum ratios appear at nearly 4.0 ms for low AR and at nearly 5.5 ms for high AR mainly due to a difference in the *M*_*y*_. The mass distributions for other ARs, the main flow to transfer the mass to the *x*-direction, and the verification of the numerical result are detailed in Supplementary Figures S1–S3, respectively.

We decomposed the height of the drop into the rim and the central film for the spherical drop and the ellipsoidal drop of AR = 1.62 in Fig. 3e and f, respectively. The rim of the spherical case is also included in Fig. 3f for comparison. Figure 3f indicates that the rim heights of the *x*- and *y*-directions show an obvious difference. The rim height of the *y*-direction only slightly increased compared with that of the *x*-direction, almost similar to the spherical case until nearly 3.5 ms; this trend is also observed in the other ARs, as shown in the inset. However, the rim height of the *x*-direction grows up more considerably than that of the *y*-direction because of the merging process of the rims and shows a clear difference between other ARs, as described in the inset. Evidently, from an increase in the rim height of the *x*-direction with the AR, the asymmetry in the mass distribution is intensified by the mass transfer from the *y*-direction to the *x*-direction as the AR increases.

**Figure 4:**
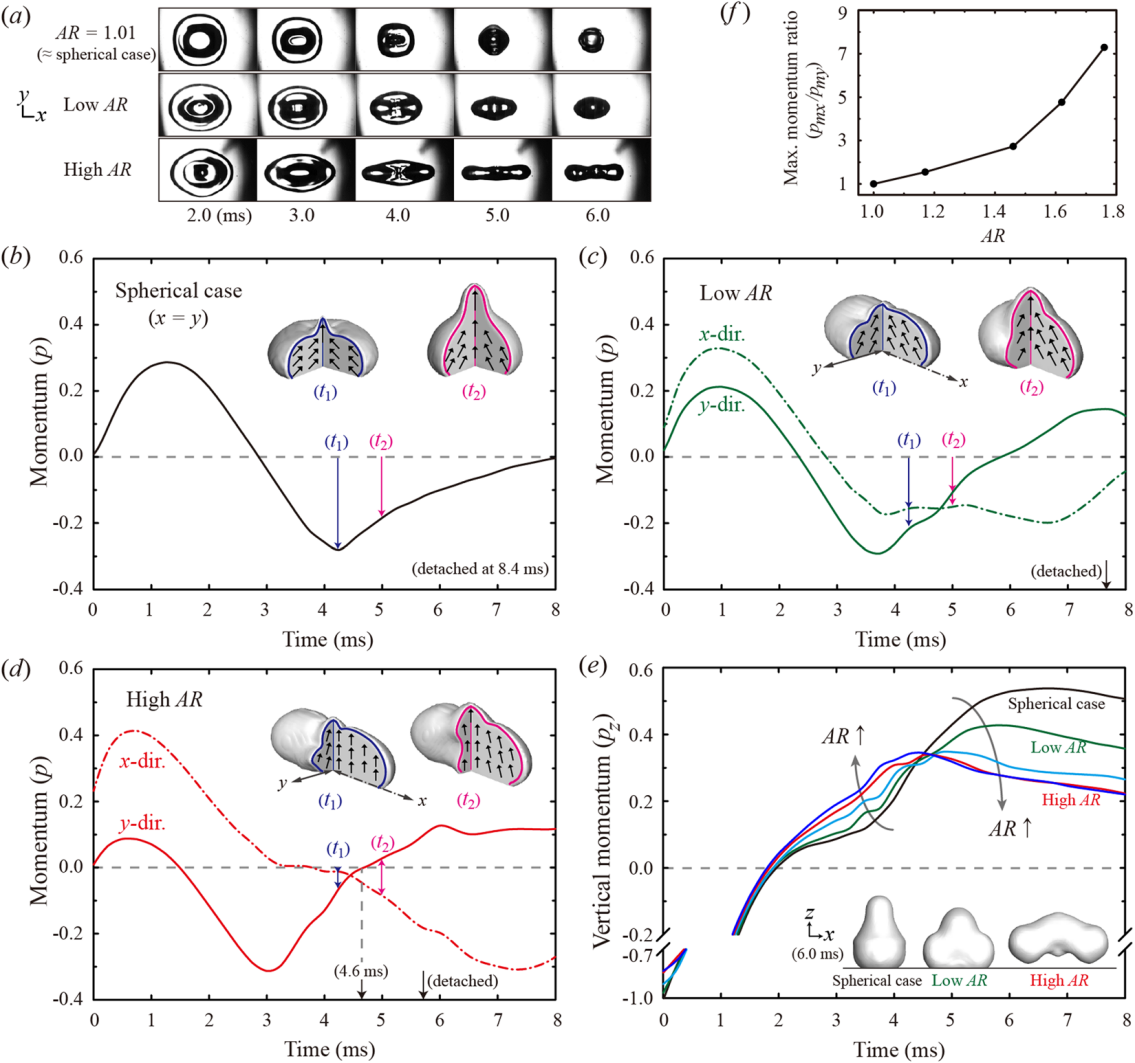
Drop retraction dynamics based on the analysis of the momentum. (**a**) Plane-view snapshots of retracting drops for the slightly deformed case (AR = 1.01) and the ellipsoidal cases (low AR = 1.12; high AR = 1.67) under We = 17. (**b**–**d**) The time variation of the momentum normalized by the initial total momentum in the principal directions for the spherical and ellipsoidal cases (low AR = 1.17; high AR = 1.62) under We = 17 based on the numerical simulation; the positive values of *p* denotes the spreading, while the negative values of *p* denotes the retracting; the insets depict the illustrations of the drop shape at specific times (*t*_1_ = 4.2 ms; *t*_2_ = 5.0 ms), and the arrows indicate the flow pattern (not to scale) in the cross-sections of the drop; the dash-dot line indicates the *x*-direction, and the solid line indicates the *y*-direction. (**e**) The time variation of the momentum along the vertical direction (*p*_*z*_) for AR = 1.00 (black), 1.17 (green), 1.46 (cyan), 1.62 (red), and 1.76 (blue); the insets depict the illustrations of the drop shape for the three cases at 6 ms; the large *p*_*z*_ is found at high AR before nearly 4 ms, but it is completely reversed thereafter. (f) The maximum momentum ratio between the principal directions (*p*_*mx*_ */p*_*my*_) as a function of AR.

To better understand how the symmetry breaking in the liquid mass distribution affects the reduction in the contact time, we focused on the retraction dynamics and shape evolution of the ellipsoidal drops. Figure 4a exhibits the plane-view images of the time-resolved shape evolution of the slightly deformed drop (AR = 1.01) and the ellipsoidal drops with low AR and high AR. The slightly deformed drop showed almost the same dynamics as that of the spherical one. The high-AR drop retracted by varying the elliptical shape and expanded further along the *x*-direction by augmenting the contrast in the liquid alignment on the direction, compared with the drop with smaller ARs. In addition, the high-AR drop first retracted along the *y*-direction by merging the rims on the *x*-direction and then retracted along the *x*-direction, whereas the slightly deformed and low-AR drops retracted simultaneously along both sides of the principal directions by converging the rims to the drop center. We suppose that the drop detachment speed can be closely related to the retraction dynamics because the asymmetric retraction of the rims alters the drop hydrodynamics significantly.

The hydrodynamics of the bouncing drop can be characterized in terms of variations of the fluid momentum for each direction based on the simulation. The momentum normalized by the initial total momentum is represented as *p*; the velocity of the momentum has a positive sign in the spreading state and a negative sign in the retracting state, which allows us to understand the net momentum along a certain direction inside the drop. Figure 4(b–d) show the time variation of the momentum in the principal directions for the spherical and ellipsoidal cases with the low AR (1.17) and high AR (1.62). The insets present the drop diagrams at two distinct times, and the arrows indicate the flow pattern in the perpendicular cross-sectional planes. The maximum *p*_*x*_ for ellipsoidal drops is always greater than the maximum *p*_*y*_, whereas that of the spherical case is placed between two values. Figure 4f represents the maximum momentum ratio between the principal directions, given as *p*_*mx*_ */p*_*my*_, where *p*_*m*_ is the maximum momentum at the initial spreading state. As shown in the figure, increasing AR results in a larger ratio.

For the spherical drop, the horizontal momentum begins to reverse its direction at nearly 3 ms and sustains the retracting state until 8 ms, as shown in Fig. 4b. The diagrams at *t*_1_ and *t*_2_ describe that the drop continually has the inward components of fluid motion, thereby causing the formation of a rising upward stream at the top of the liquid. The vertically elongated drop is detached from the solid once the horizontal momentum is close to zero. For the low-AR drop, the *p*_*x*_ begins to reverse its direction at nearly 3 ms and maintains the retracting state until 8 ms, whereas the *p*_*y*_ moves back toward the spreading state from the retracting state before the drop detachment, as shown in Fig. 4c. The temporal variations in *p*_*x*_ and *p*_*y*_ for the low-AR drop are notably different from those of the spherical case. Figure 4d showing the high-AR drop indicates that the *p*_*x*_ remains almost zero at *t* = 3–4 ms when the liquid alignment is being formed as the merging of the rims along the *x*-direction occurs (high AR of Fig. 4a). In addition, when the alignment is made, the *p*_*x*_ and *p*_*y*_ switch directions at *t* = 4–5 ms, which is considerably reduced. This finding implies that the vertical momentum is more pronounced than the horizontal momentums, as depicted in the diagrams at *t*_1_ and *t*_2_, showing a striking feature of the high-AR drop. The diagrams exhibit that the slight inward motion of fluids in both principal directions is found at *t*_1_, whereas the slight inward motion in the *x*-direction and the slight outward motion in the *y*-direction are observed at *t*_2_.

The temporal variation in the vertical momentum (*p*_*z*_) for several ARs is plotted in Fig. 4e. The plot shows that the vertical momentum is enhanced with increasing AR at *t* = 2–4 ms. This implies that most of the liquid participates in an upward motion for the high-AR drop, compared with the spherical and the low-AR drop, thereby contributing to fast drop detachment that can be closely related to the momentum transfer rate. Careful observation of the high-AR drop in Fig. 4(d,e) reveals that *p*_*y*_ at the retracing state is close to zero at 4.6 ms, and *p*_*z*_ reaches its maximum value at the same time, whereas *p*_*x*_ remains almost unchanged to zero. This finding indicates that the inward motion in the *y*-direction is mainly used to increase the upward motion in the vertical direction at *t* = 3–5 ms when the liquid alignment is ongoing, thereby causing the momentum transfer from *p*_*y*_ to *p*_*z*_; indeed, the momentum transfer to the vertical direction almost finishes with the momentum in the *y*-direction (*p*_*y*_ → 0) at 4.6 ms, corresponding to relatively earlier time, compared with the low-AR cases; thus the *y*-direction can be regarded as a leading principal direction of the liquid alignment that adjusts the rate of the momentum transfer. As the next step after 4.6 ms, the capillary retraction of the liquid alignment initiates the shape oscillation, which results in a decrease in *p*_*x*_ (contraction), an increase in *p*_*y*_ (expansion), and a slight decrease in *p*_*z*_ (mainly due to the increased non-axial momentums), as shown in Fig. 4(d,e).

Distinct from the high-AR case, *p*_*z*_ of the spherical and low-AR drop is less pronounced than the horizontal momentums at *t* = 2–4 ms in Fig. 4e, causing the liquid to move rapidly toward the drop center that remains stationary until enclosed with the liquid elongated vertically. After nearly 4 ms, however, the spherical and low-AR drops show a strong increase in *p*_*z*_ because the horizontal momentums are massively transferred to *p*_*z*_. The increased amount of *p*_*z*_ is mainly used to deform the drop shape into the liquid column, as described in the diagrams. The preferential momentum transfer to *p*_*z*_ occurs spatially from the upper part of the drop due to rising liquid squeezed out by the rims that are moving inward almost all the way.

**Figure 5:**
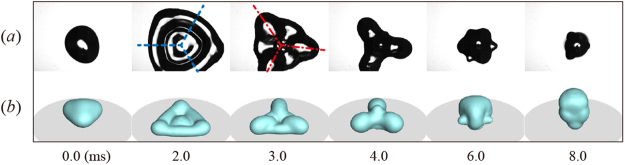
Impacting dynamics of a drop with initially three-lobed shaping for We = 20, based on (**a**) the experiment and (**b**) numerical simulation. During the retracting process, the liquid is aligned on the three principal axes and released along the axes by the capillary retraction, which leads to the formation of the liquid column that is still in contact with the bottom surface; the blue dashed lines represent a set of the principal axes for the initial retraction (indicated at 2.0 ms) and the red dash-dot lines represent a set of the principal axes for the liquid alignment (indicated at 3.0 ms); the drop detached from the surface within 8.9 ms, observed experimentally.

After nearly 5 ms, the *p*_*z*_ of the spherical and the low-AR drops continually increase and reach the maximum values, whereas that of the high-AR drop steadily declines, even with the lowest value among several ARs. This means that the horizontal momentum of the spherical and the low-AR drops is still being transferred into the vertical direction by the almost symmetric retracting behavior, compared with the high-AR drop. Two factors significantly affect the efficient drop detachment of high-AR drops: one is to have a uni-axial retraction in overall dynamics, which leads to liquid mass to be aligned and then released along the *x*-direction, and the other is to retain a small inertia of rims to be merged when the alignment is ongoing (see *M*_*y*_ of Fig. 3d). To validate a role of the uni-axial (asymmetric) retraction, we conducted the additional experiment by initially deforming the drop into a three lobed shape using the modified ring electrodes^27^, which was reproduced by the simulation. Figure 5 shows that the liquid was aligned on a set of the principal axes for the liquid alignment (denoted as red dash-dot lines at 3.0 ms) and released along the axes by the capillary retraction, which led to the formation of the liquid column that was still in contact with the bottom surface; the blue dashed lines represent a set of the principal axes for the initial retraction, as shown at 2.0 ms. The experiment showed that drop finally bounced off the surface within 8.9 ms, resulting in little reduction in the contact time, which indicated that the symmetric retraction along the principal axes would prevent the improvement in the resident time.

Accordingly, enhancing the asymmetry in the mass and momentum distribution plays a key role in promoting the drop detachment. Moreover, the retraction of the liquid alignment for high AR can possibly assist the upward motion for bouncing by motivating the drop to restore a spherical shape, because the capillary pressure is inherently induced by a high curvature occurring at the tips of both ends along the *x*-direction, as shown at 5 ms in Fig. 4a. In particular, we found that the “zip-up” process for the liquid alignment has a notable ability to spatially adjust the momentum transfer from *p*_*y*_ to *p*_*z*_; the momentum and mass distributions in the alignment process are detailed in Supplementary Figure S4.

The vertical momentums of the highly deformed drops with AR = 1.62 and 1.76 show minimal difference, as shown in Fig. 4e, which is consistent with our experimental result that the contact time and maximum bounce height increase with the AR but has no significant variation at AR > 1.5. The result means that the extremely deformed ellipsoidal shape only slightly affects the change in the mass and momentum distributions. The relationship can be associated with the surface energy of the liquid alignment, which is transferred mostly to the non-axial KE due to the shape oscillation of the bouncing drop. The drop oscillation enables an exchange of the non-axial KE between the principal directions; the amplitude of the oscillation showed no significant increase for the two ARs, which also confirmed the effect of AR on the bounce magnitude.

In conclusion, we have proposed a method to control the bouncing drops on a superhydrophobic solid surface by deforming them into ellipsoidal shapes at the moment of impact and confirmed the feasibility through experimental and numerical studies. The ellipsoidal drop shape alters the bouncing behavior significantly, providing a remarkably efficient pathway to reduce the contact time and maximum bounce height below the spherical one by at least 30% and 60% when AR > 1.5, respectively. In particular, the extraordinary rim dynamics for high AR during the retraction produces the liquid alignment, which leads to the symmetry breaking in the mass and momentum distribution. The quantitative study of the temporal variation in the momentum shows that, while the liquid alignment is being formed, the momentum in the *y*-direction at the retracting state is considerably transferred into the vertical momentum that induces an upward motion for the prompt drop detachment. The methodology forms a striking contrast to the spherical drop impact showing the retarded momentum transfer to the vertical direction and the consequent formation of the liquid column.

The hydrodynamic features of the ellipsoidal drop impact will provide insights into shape-dependent impact dynamics, including a reduction in the resident time and the bounce magnitude on non-wetting surfaces. The scenario in drop impact might demand an effective modulation of the aspect ratio to secure controlling ability to stay dry, self-clean, and prevent icing, which could be accomplished by deforming a charged drop into ellipsoids under modulated electric fields or exerting the air pressure on the drop around the impact. The challenging shape-dependent drop impact suggests a new avenue for the control of its deposition in industrial applications, such as pesticide spraying, surface cleaning, and spray cooling.

## Methods

### Experimental Setup

Ellipsoidal drops were generated using a conventional nozzle-ring electrohydrodynamic device. A flat stainless-steel nozzle (Hamilton, 27 gauge) and a ring made of copper with 20 mm outer diameter and 2 mm thickness were used. The nozzle and ring were spaced approximately 2 mm apart. The inner hole of the ring has an elliptical shape, which can exert a stronger electric field in a minor axis than a major axis of the ellipse. A dc voltage of 6–7 kV was applied as a pulse signal between the electrodes for 7–10 ms. Before the voltage was applied, we produced a distilled water drop with our prescribed volume (~4 *μ*L) at the end of the nozzle by a syringe pump and kept it stationary until the voltage was applied based on the hanging-drop method^30^. The effective drop diameter (*D*) was nearly 2 mm. When the voltage was applied, the drop started to stretch vertically and then was detached from the nozzle. The drop deformed into a non-axisymmetric ellipsoidal shape around the ring, which induced a shape oscillation before touching the solid. The duration time of the pulse signal was set so that the drop deformation could be maximized and the effect of the electric field on the oscillation could be suppressed. The spherical drop was produced non-electrostatically by dripping from the nozzle. The overall bouncing behavior was recorded by using two synchronized high-speed cameras (Photron, Fastcam SA3) to obtain side-view snapshots in perpendicular planes and plane-view snapshots. Several impact speeds were obtained by adjusting the falling height. To vary the AR at a constant impact speed, we adjusted the falling height slightly or the space between the electrodes slightly. The amount of the drop charge was measured as 0.30 ± 0.01 nC by collecting the falling drop into a Faraday cup linked to an electrometer (Keithley 6514). The electric charge only slightly affected the impact dynamics of the ellipsoidal drop impact, based on our previous experimental study showing that the overall impact behavior is comparable to the simulation result of neutral drops. The superhydrophobic surface was fabricated by the functionalization of an aluminum plate through both etching with sodium hydroxide and grafting with fluoroalkylsilane^31^. We first grinded the aluminum plate with sandpaper 500, 800, and 1000 to roughen the plate. Then, we immersed it in a mixture of 0.1 mol/L NaOH and 0.04 mol/L FAS for approximately 30 min and rinsed it off. To get rid of ethyl groups, FAS was mixed with NaOH aqueous solution, which induced the silane group to react with the plate submerged in the solution. The contact angle was measured as 160 ± 3° by using a sessile drop method including a dynamic drop test with the high-speed camera for advancing and receding angles. To obtain binary plane-view images, we made transparent superhydrophobic surfaces in the way that a clean glass slide was treated with 95 *μ*L methyltrichlorosilane and 0.25 mL concentrated hydrochloric acid, which formed polymethylsiloxane network acting as air pockets^32^; this yielded the contact angle of 155 ± 3° measured in the same manner as the aluminum sample. All the quantitative studies of the drop dynamics were carried out by using the aluminum sample, and the dynamics was analyzed from two synchronized side-views, although two sample surfaces showed only slight difference in the dynamics.

### Numerical method

The incompressible, unsteady, and three-dimensional numerical simulation using VOF method was conducted to rationalize the impact dynamics of ellipsoidal drop impact. In the simulation, the liquid and vapor were used as water and air at room temperature, respectively. The mathematical model of the prolate ellipsoids was adopted as *x* ^2^/*a* ^2^ + *y* ^2^/*b* ^2^ + *z* ^2^/*a* ^2^ = 1, which was set to the initial phase condition for liquid, where *a* and *b* are the minor and major axes of the horizontal planes, respectively. The *x* and *y* axes were the principal axes in the horizontal planes, and the *z* axis was the vertical axis normal to the solid. Although an accuracy of the interfacial curvature of the VOF model is known to be relatively low compared to other multiphase flow methods, the model has been considered suitable for the analysis of the droplet impact because of its advantage in conserving the volume. The numerical methods, including boundary conditions and numerical schemes, were based on studies on the droplet impact on the solid surface using VOF^33,34^. The mesh resolution of the drop was 40 cells per diameter (~2 mm) and was dense near the solid in a rectangular domain. A time step and maximum internal iteration were set to 1 *μ*s and 30 per time step, respectively, which enabled normalized residuals to be less than 10^–5^. A contact angle of 160° was employed, which can reasonably predict the spreading and retracting dynamics. The calculation of the mass per length and the momentum of the drop over time were conducted through a surface integral of the density in a cross-sectional area of the drop and a volume integral of the density multiplied by the velocity component in a certain direction for the drop, respectively; a sign of the velocity component was defined so that a positive sign is in the spreading state and a negative one in the retracting state.

## Electronic supplementary material

Supplementary materials
