# Supplementary material for: Bouncing of an ellipsoidal drop on a superhydrophobic surface
Source: Sci Rep. 2017 Dec 18;7:17699. doi: 10.1038/s41598-017-18017-2 (PMC5735163; doi:10.1038/s41598-017-18017-2)
Supplement: Supplementary file 1 — Supplementary materials [file 41598_2017_18017_MOESM1_ESM.pdf]

Bouncing of an ellipsoidal drop on a superhydrophobic surface

Sungchan Yun*

Department of Mechanical Engineering, Korea National University of Transportation, 27469, Republic of Korea

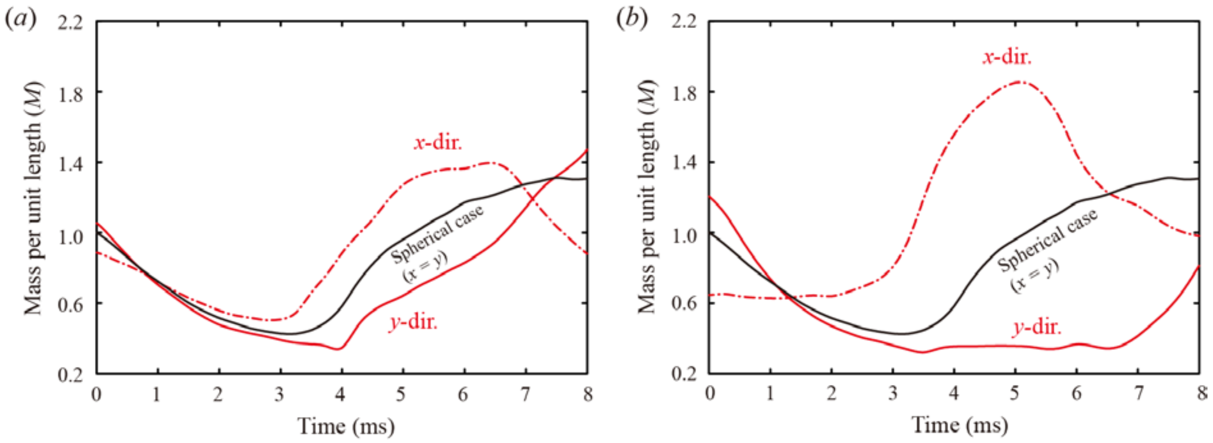

Fig. S1. Temporal evolution of the normalized mass per unit length (M) along the cross-sectional planes (indicated in Fig. 2b) for (a) AR = 1.17 and (b) AR = 1.76; the mass per unit length is scaled by that of the spherical case at the initial state (t = 0); As for red lines, the dash-dot line indicates the x-direction, and the solid line the y-direction. The discrepancy of the mass between the principal directions increases as the AR increases after nearly 3ms.

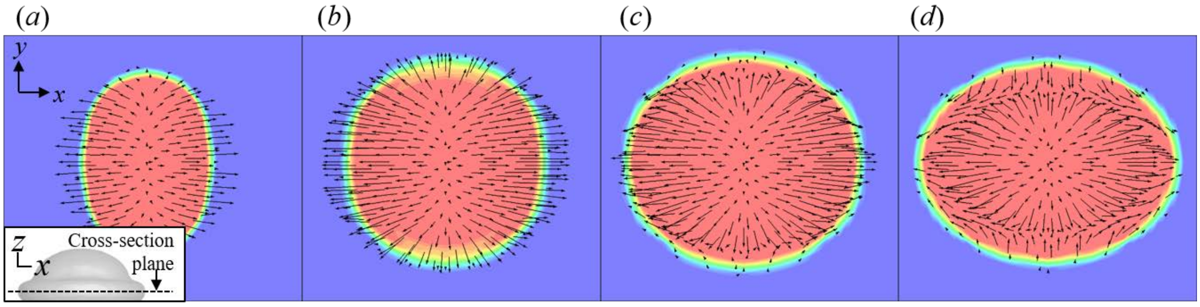

Fig. S2. Numerical result of the velocity field (arrows) and phase distribution [contour: liquid (red); air (blue)] in the x-y plane, at a distance of 0.3 mm from the plate, indicated in the inset of (a); time elapses from (a) to (d) at 0.5 ms intervals, after the ellipsoidal drop starts to impact at 0 ms with AR = 1.62 and We = 17. The predominant component of the x-direction in the main flow induces the mass transfer from the y-direction to the x-direction during spreading, leading to the formation of the liquid alignment.

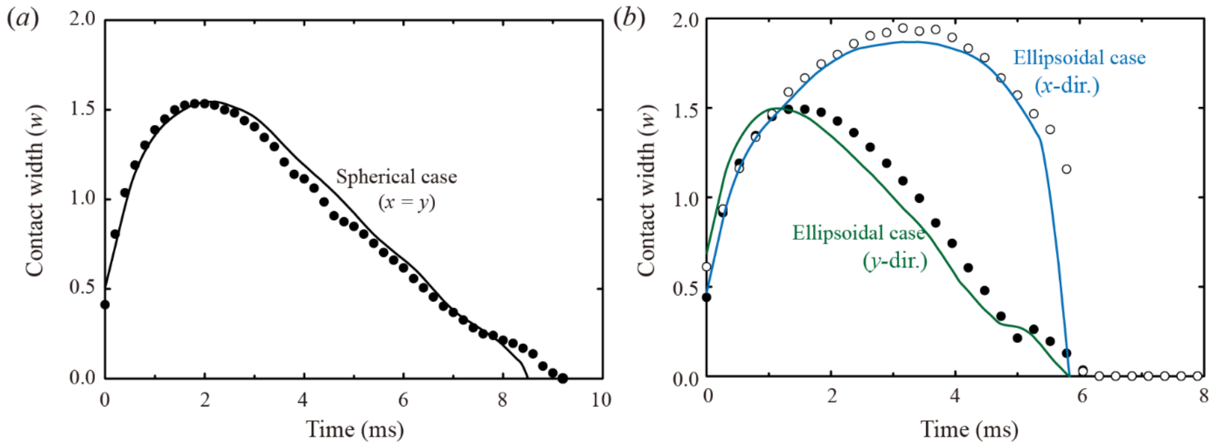

Fig. S3. Verification of the numerical model based on the normalized contact width of (a) spherical case and (b) ellipsoidal case at AR = 1.62 shows a reasonable prediction of the impact dynamics; the symbols represent the experimental data, and the line the numerical data.

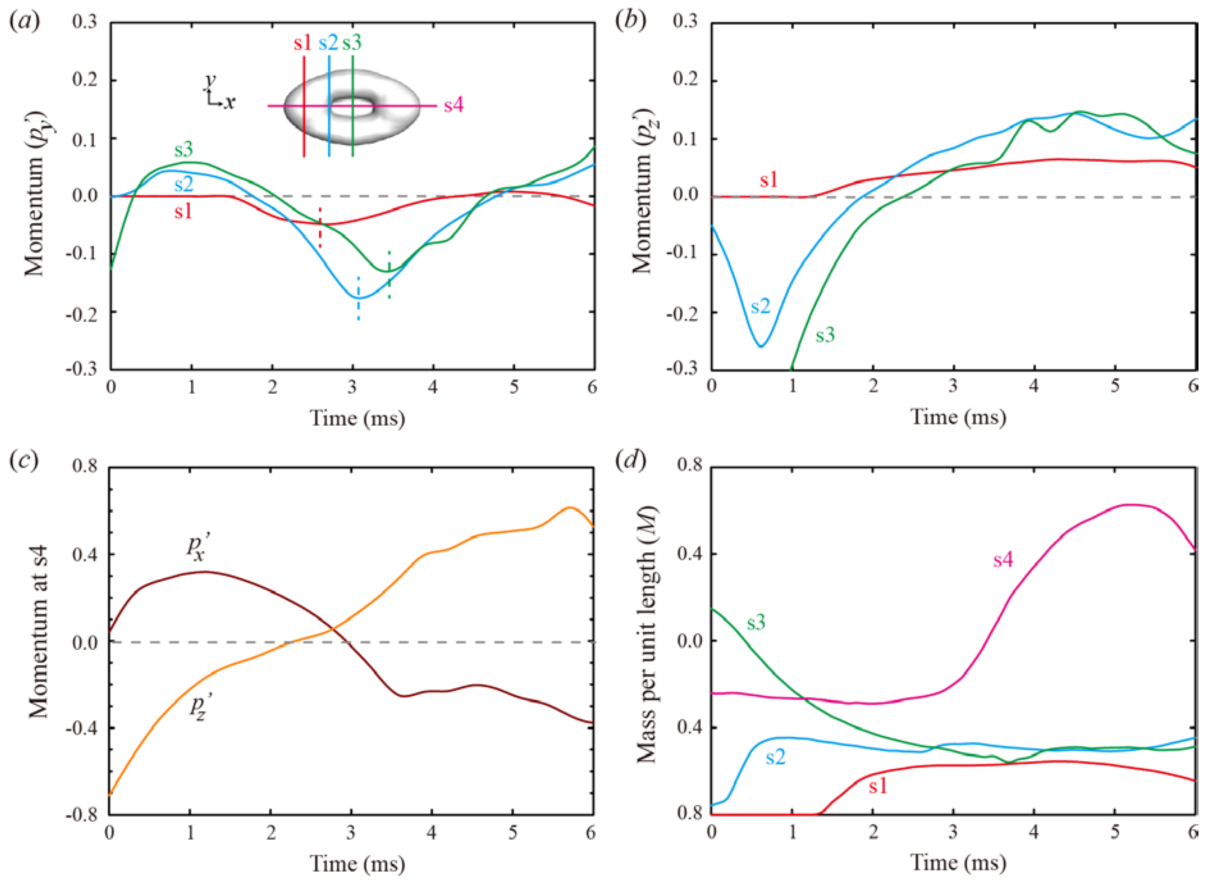

Fig. S4. Temporal variation of the momentum and mass distribution in several cross-section planes (s1–s4) shows that the “zip-up” process for the liquid alignment can spatially adjust the momentum transfer from the *y*-direction to the *z*-direction. (a–c) The normalized momentum per unit lengths (*p*’) in the cross-sections, s1–s4, are obtained based on the numerical simulation; the momentum per unit length denotes the surface-integral of the density multiplied by the velocity for the liquid and is scaled by the initial value for the spherical case; the positive value of *p*’ corresponds to the spreading state, whereas the negative value the retracting state; the s1, s2, and s3 are space apart at 0.8 mm, and the vertical dashed lines indicate the maximum values at the retracting state, as shown in (a); (d) Temporal evolution of the normalized mass per unit length (*M*) along the cross-sectional planes shows that the liquid alignment has almost uniform mass distribution along the *x*-direction after nearly 3ms, whereas the *M* obtained in s4 shows a strong increase; the mass per unit length is scaled by that of the spherical case at the initial state (t = 0).
